# Proteasome Activation to Combat Proteotoxicity

**DOI:** 10.3390/molecules24152841

**Published:** 2019-08-05

**Authors:** Corey L. Jones, Jetze J. Tepe

**Affiliations:** Department of Chemistry; Michigan State University, East Lansing, MI 48824, USA

**Keywords:** proteasome, activation, enhancement, intrinsically disordered proteins, IDP, neurodegenerative disease, proteotoxic, oxidative damage, aggregates

## Abstract

Loss of proteome fidelity leads to the accumulation of non-native protein aggregates and oxidatively damaged species: hallmarks of an aged cell. These misfolded and aggregated species are often found, and suggested to be the culpable party, in numerous neurodegenerative diseases including Huntington’s, Parkinson’s, Amyotrophic Lateral Sclerosis (ALS), and Alzheimer’s Diseases (AD). Many strategies for therapeutic intervention in proteotoxic pathologies have been put forth; one of the most promising is bolstering the efficacy of the proteasome to restore normal proteostasis. This strategy is ideal as monomeric precursors and oxidatively damaged proteins, so called “intrinsically disordered proteins” (IDPs), are targeted by the proteasome. This review will provide an overview of disorders in proteins, both intrinsic and acquired, with a focus on susceptibility to proteasomal degradation. We will then examine the proteasome with emphasis on newly published structural data and summarize current known small molecule proteasome activators.

## 1. Introduction

Inhibitors of the proteasome have long held clinical significance, particularly in the treatment of multiple myeloma (MM). Despite past successes, improvement of current inhibitors is still an active area of research with widespread interest in expanding the scope of treatable ailments ranging from arthritis to autoimmune disorders [1,2,3,4,5].

In contrast, research on the enhancement of proteasome activity has lagged behind, despite being discovered first [6]. Lack of progress in this field in the four decades since the discovery of the proteasome [7,8] may be attributed to a variety of factors, including the complexity of proteolytic enhancement (vide infra) and general lack of biochemical tools. However, advancements in our understanding of protein homeostasis (proteostasis), the proteostasis network (PN), and its central role in maintaining cellular health is bringing proteasome enhancement research to the forefront. Recent reviews thoroughly cover the PN’s role in aging [9,10], cardiac health [11], neurodegenerative disease [12,13], and other diseases derived from PN dysfunction [14,15]. 

In brief, the proteostasis network (PN) is a highly intricate system tasked with maintaining the fidelity of over 10,000 proteins [16], a herculean task requiring the coordinated action of synthesis machinery (ca. 280 components [17]), chaperones (ca. 330 components [18]), and degradation machinery (ca. 1400 components [19]). An abundance of degradation machinery is needed, as the proper folding of polypeptides is inherently error prone, owing to the vast conformational space available [20]. The crowded intracellular space, with a concentration of macromolecules at nearly 300 g/L, only exacerbates the problem [21]. 

The loss of proteome fidelity leads to an accumulation of non-native protein aggregates and oxidatively damaged species, hallmarks of an aged cell [22]. These misfolded and aggregated species are often found, and suggested to be the culpable party, in numerous neurodegenerative diseases including Huntington’s, Parkinson’s, Amyotrophic Lateral Sclerosis (ALS), and Alzheimer’s Diseases (AD) [23,24,25]. 

Numerous reasons have been put forth for the age-related decline of the PN [26]; however, it may be succinctly stated as small mutations over long time periods causing decreased efficiency of the regulation machinery. Further, damage to cellular content is commonplace, as air pollution [27,28], UV radiation [29], and natural cellular processes [30,31] incur an oxidative penalty. Eventually, this overcomes the proteasome’s capacity for clearance, leading to the accumulation, oligomerization, and aggregate formation of some proteins. 

Many strategies for therapeutic intervention in proteotoxic pathologies have been put forth [24]; one of the most promising is bolstering the efficacy of the proteasome to restore normal proteostasis. This strategy is ideal, as monomer precursors and oxidatively damaged proteins, so called “intrinsically disordered proteins” (IDPs), are targeted by the proteasome. 

This review seeks to be a reference and update of the field while affording a general review of the current state of proteasome activation research. To provide an accurate picture, we will overview disorders in proteins, both intrinsic and acquired, with attention to their susceptibility to proteasomal degradation. We will then examine the proteasome with emphasis on newly published structural data. The final section will then cover current known synthetic activators of the proteasome, and we will conclude with a discussion of the current barriers to progress of the field. 

## 2. Intrinsically Disordered Proteins

For a detailed discussion, the reader is directed to the many excellent and recent reviews available on IDP function [32,33,34], role in cellular signaling [35,36,37,38], advantages in protein-protein interactions [39], and regulation and disease [40,41,42,43]. Reviews of empirical studies on intrinsically disordered proteins using single molecule methods [44], NMR spectroscopy [45], methods of characterizing conformational ensembles [46], and identification [47] are available and will not be discussed here. This section will simply define and present key points of the structure and function of IDPs, with select examples, and then discuss the similarity of oxidatively damaged proteins before highlighting their roles in disease states. 

### 2.1. Defining “Disorder”

Understanding IDPs as a broad class of functionally important proteins began in earnest in the mid-1990s with a bioinformatics study on the emerging complete genome sequences. Analysis revealed that disordered regions were actually common in eukaryotic proteins [48], with some estimates proclaiming that 44% of human protein-coding genes have disordered segments of at least 30 residues [49]. Today it is known that IDPs play indispensable roles in numerous cellular processes like signaling, transcription/translation, and cell cycle progression [35,38,50,51,52]. The recent flux in IDP research publications would suggest that these complexes are a recent discovery; yet, the reality is that IDPs have been reported periodically over the past 80 years [53,54].

A plausible explanation of this inconsistency, proposed by Uversky [55], is a traditional lack of a unifying terminology. IDPs have been previously described as floppy, pliable, flexible, partially folded, natively denatured, etc. [34]. It has also been suggested that the term IDP is not ideal; however, it is the currently recognized umbrella term found in the literature. A comprehensive discussion on classification is available [56]. The accepted definitions, derived from common use in the field, may be listed as follows [56,57,58]:

- Any functional protein or protein domain possessed of a unique 3D structure described by minimal fluctuation around their equilibrium Ramachandran angles is termed a “structured protein”.
- Any functional protein or protein domain that exists as a dynamic ensemble lacking specific equilibrium Ramachandran angles with backbone atomic positions that naturally undertake non-cooperative conformational changes is termed an “intrinsically disordered protein or region”.

Great effort has been put forth in classifying IDPs. The development of Dunker and co-workers original 28 functions of IDPs [59] has resulted in 6 distinct IDP and intrinsically disordered protein region (IDPR) functions being in common usage [56]. A single protein may belong to several classes simultaneously [60], and broader categories are also used [61]. A comprehensive discussion about intrinsically disordered proteins detailing function, structure, and nomenclature was recently published by van der Lee and coworkers [56], and is highly recommended. 

### 2.2. IDP Structure and Function 

**Figure 1:**
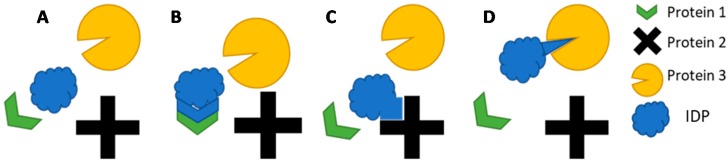
Cartoon of IDP molding itself to accommodate differently shaped binding pockets to elicit some response.

IDP/IDPRs are characterized by low sequence complexity and biased amino acid composition (preference for highly charged and hydrophilic residues) [58]. The lack of hydrophobic/bulky residues results in a relatively flat energy surface and existence as a structural “ensemble” of interconverting conformational states [62]. Disordered regions leverage their high conformational freedom to maximize potential binding partners (Figure 1). Specificity is generally achieved per partner by multiple low affinity contact points, and the entropic loss keeps the binding interaction transient. 

**Figure 2:**
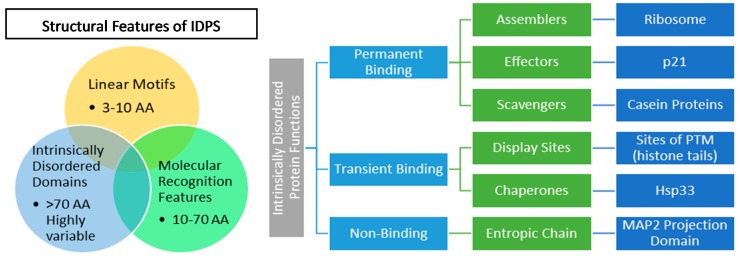
Venn diagram depicting three common features found in IDP structures and their complex interrelationship (Left). Hierarchy showing six IDP functions (green) from three binding modes (light blue) with an example of each type (dark blue, Right).

The size of the disordered region plays a crucial part in determining the type of binding interaction, and therefore function, of the IDP. Disordered regions are divided broadly into three main types of recognition elements: linear motifs [63,64] (LMs, 3–10 residues), molecular recognition features [65,66] (MoRFs, 10–70 residues), and intrinsically disordered domains [67,68] (IDD, Figure 2). 

Short linear motifs (LMs/SLiMs/MiniMotifs) are perhaps the most common functional feature within IDPs. Aided by the flexible nature of the surrounding disordered protein, LMs bind mainly to the surface of globular domains. However, as LMs provide a very small surface area to bind to (~3–10 AA), they often depend on multiple low affinity interactions to elicit action. LMs are also used to herd proteins to certain subcellular locations and are found most often in use as dynamic signaling elements. Microtubule-associated protein 2 (MAP2) projection domain is a common example of a non-binding functional protein. MAP2 provides spacing in the cytoskeleton by repelling molecules encroaching on microtubules [69].

As the disordered domain gets larger, the increase in surface area of binding begins to compensate for the loss of freedom and binding time increases. Molecular recognition features (MoRFs) are larger domains of disorder and promote specific protein-protein interactions. MoRFs undergo disorder-to-order transition on binding and are generally found to be biased toward the bound conformation in solution. MoRFs are classified according to the secondary structure they adopt upon binding to their partner protein: α-MoRF, β-MoRF, and ι-MoRFs for alpha helices, beta sheets, and irregular (though rigid) structures, respectively. A common example of MoRF binding domain is found in p53, which contains an α-MoRF (residues 40–60) that acts as a secondary binding site for Mdm2, as well as a primary binding site for RPA70 [70]. 

Intrinsically disordered domains (IDDs) are the largest disordered domains identified. One example includes the kinase-inhibitory domain (KID) of Cdk inhibitors (i.e. p27 [51]), which have been shown to be fully disordered in solution. 

The main functional features described above exist on a continuum though may be mutually exclusive. As such, a single protein can be comprised of multiple disordered regions that belong to different functional classes, offering immense conformational variability and adaptability. These functional features conspire to dictate binding interaction, and therefore function, of IDPs. These binding interactions are broadly divided into three categories: non-binding, transient, and permanent binding, which are further divided by specific function (Figure 2 illustrates this tiered system and provides some examples).

IDPs are structurally well suited to be sites of post-translational modifications [41] and signal potentiation [71]. They are often found as, or closely associated with, hubs within protein networks [72,73,74] and chaperones (though some debate seems to still be present [75,76,77]). This is exploited by the cell to facilitate regulation through varied PTMs, and recruitment/localization of different binding partners. 

IDPs play critical roles in the regulation of numerous cellular processes. Unsurprisingly then, dysregulation of their activities is heavily implicated in a variety of disease pathologies.

### 2.3. IDPs and Neurodegeneration

We will focus on IDPs associated with neurodegenerative diseases (NDD) as it is more germane to later discussions; however, IDPs are found in numerous other disease states and the reader is encouraged to pursue the literature in those fields including: cancers [72,78,79,80,81,82], diabetes [83,84], and more [41,85]. 

#### 2.3.1. Neurodegeneration

Neurodegeneration is characterized by the loss of structure or function of neuronal cells. The deterioration of neurons, which are not readily regenerated, leads to dysfunction and disability over time. In general, this is a slow process; it can be months or years before symptoms are prevalent enough for diagnosis. While dozens of NDDs are known, each with a unique symptom presentation, NDDs are unified in that each is the result of dysfunction in different regions of the central and peripheral nervous system [36]. Further, this dysfunction is well established to be due to proteotoxic signaling of oligomerized, misfolded, or aggregated species [86,87]. Literature reviews on this subject are plentiful [23,86,88,89,90,91,92], cataloging well over 100 central nervous system (CNS) diseases and levying accusations of culpability at an almost equally numerous numbers of IDPs. We will limit our discussion to a few illustrative examples of the most commonly implicated IDPs. 

#### 2.3.2. α-synuclein 

α-synuclein is perhaps the most studied disease-related IDP [93,94,95]. α-synuclein is very sensitive to its environment, possessing a wide variety of unrelated conformations [96]. It is approximately 140 amino acids in length encoded by the SNCA gene. At physiological pH, α-synuclein is almost fully disordered though has been reported in various partially folded states [97,98]. The function of α-synuclein is not well understood, but it is primarily found in the brain, accounting for up to 1% of all protein in the cytosol of brain cells [99]. Studies have shown that α-synuclein is mainly found in the presynaptic termini of neurons [100], where it may function as a molecular chaperone for SNARE complexes [101]. However, as mentioned in the preceding section, as an IDP, α-synuclein would be expected, and has been shown, to play numerous roles through a variety of interactions. Research has demonstrated that α-synuclein has significant interaction with tubulin [102], presents with linker-like behavior [103], blocks endoplasmic reticulum-to-Golgi trafficking [104], plays an essential, albeit unknown, role in memory [105], and even displays potential antioxidative behavior through interaction with phospholipids among others [106].

Diseases that result from the accumulation of oligomerized species of α-synuclein are collectively termed synucleinopathies. Various aggregates of α-synuclein, termed Lewy-bodies, are hallmarks of these disease states. The most common of these is Parkinson’s disease (PD), a complex chronic and progressive NDD.

#### 2.3.3. Amyloid β (Aβ) and Tau 

Damage to the brain by Alzheimer’s disease (AD) progresses for 10–20 years before symptoms become apparent. AD is the most common cause of dementia world-wide (over 46 million afflicted), and no significant breakthroughs in treatment options have occurred in the past twenty years [107].

Hallmarks of AD are the presence of two types of insoluble protein aggregates: extracellular amyloid deposits and intracellular neurofibrillary tangles (NFTs) [108]. Aβ, a 40–42 residue peptide, comprises the bulk of amyloid deposits while a hyperphosphorylated version of tau protein are found within the NFTs [109]. Each of these proteins are structurally disordered, with tau actually deriving function from its disordered nature [110], and great effort has been put into understanding causative and mechanistic aspects of their dysfunction and aggregation [111,112,113]. Despite the consensus that these IDPs are key components of the disease’s pathology, it is the oligomeric forms of these IDPs that are the likely neurotoxic species in these disorders [114].

It is also worth noting that tau is implicated in a host of other NDDs, collectively known as tauopathies, including motor neuron disease with NFTs, argyrophilic grain disease, myotonic dystrophy, and many others [115,116].

#### 2.3.4. Prion Proteins

Prion Protein (PrP) is another amyloid-forming protein that induces neurodegeneration [117,118,119,120]. Cellular PrP undergoes a conformational change to an insoluble form known PrP^Sc^ and is believed to be the causative agent in transmissible spongiform encephalopathies (TSEs), such as Creutzfeldt-Jakob-Disease (CJD). TSEs constitute a class of fatal mammalian NDDs which are unique in that they are transmissible. The last 100 residues of the N-terminus are unstructured, and the literature suggests that it is within this IDPR that plays key roles in gain of toxicity for prion propagation and NDD [47,121]. 

#### 2.3.5. Polyglutamine Repeats 

Polyglutamine repeats (polyQ) are disordered by nature with healthy individuals expressing between 16 and 37 residues [122,123,124]. Disease states are found most prevalently when expression exceeds 38 residues. The prevailing theory is that the IDPR generated by polyQ increases in tendency to aggregate with increasing sequence length [125,126,127,128,129]. To date, multiple NDDs have been identified as arising from overexpression of polyQ with Huntington’s disease (HD) being the most well-known [124]. 

### 2.4. Acquired Disorder: Oxidative Damage

Oxidative stress, the production of non-native reactive species that effect the redox potential of the cell, is a natural byproduct of aerobic metabolism [31,130]. Reactive oxygen species are able to damage cellular structures, proteins being a primary target due their status as the most abundant macromolecule of mammalian cells [131]. 

**Figure 3:**
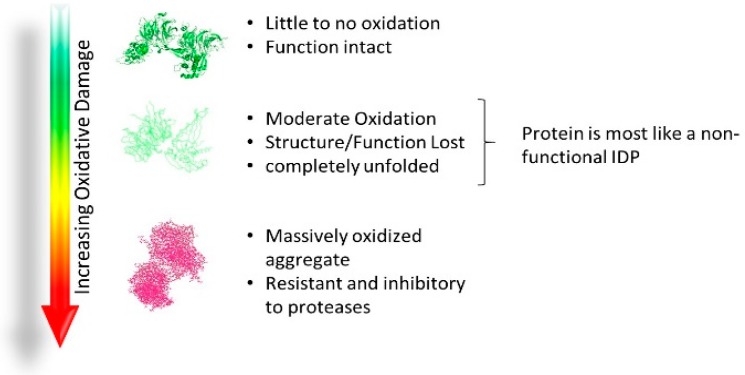
Processive increases in oxidation and the effect on structure and function of proteins from intact function to proteolytic resistant and aggregated complexes (simplified).

Despite the utility of disordered regions and proteins, all increases in disorder are not necessarily beneficial. Structured proteins require a defined 3D shape to maintain proper function. Oxidative damage decreases the hydrophobicity of the structure, the key driving force of protein folding [132,133,134], leading to increased solubility and unfolding of the protein. As oxidation increases (Figure 3), the conformational landscape of the protein begins to flatten and resemble an IDP ensemble. 

Eventually, excessive protein oxidations can result in protein cross-linking and the formation of insoluble aggregates. En route to this insoluble fate, overly oxidized proteins precipitate numerous other stresses to the cell, and are strongly implicated in the aging process [26,135,136]. Excessive oxidation is also implicated as a hastening factor in neurodegenerative disease as it has been shown that membrane bound α-synuclein may use oxidized lipid membranes as nucleation sites for aggregation [100]. Finally, oxidized proteins may catalyze the oxidation of other proteins spawning more instances of these issues and inhibiting endogenous pathways for their removal [137,138,139].

Aggregates, once formed, undergo clearance utilizing the lysosome. The processes associated with this pathway are termed collectively autophagy, which has recently been reviewed [140,141]. Yet, despite its obvious importance, autophagy largely operates non-selectively and relatively slowly; requiring a more proactive proteolytic pathway to address immediate proteotoxic stresses [26,142,143,144,145,146,147,148,149,150].

Preventing the formation of oligomerized and/or aggregated IDPs and oxidatively damaged proteins is the task of the proteasome through two major and complementary pathways: the ubiquitin dependent proteasome system (UPS) and the ubiquitin independent proteasome system (UIPS) [149,151,152,153,154,155].

## 3. Proteasome Systems

The importance of the proteasome is difficult to overstate, as the cell has evolved to utilize its proteolytic power in several specialized tasks including cell cycle regulation, differentiation, the inflammatory response, immune function, and apoptosis [156]. The proteasome comprises between 1% and 2% of a healthy cell’s proteome [157], and is found within all kingdoms of life. Because excellent detailed reviews of the proteasome’s architecture [158], biological assembly [159], and its role in human health [160] are available, this section will merely outline the proteasome and associated pathways before discussing the gating mechanism of this protease in detail. 

### 3.1. General Structure 

**Figure 4:**
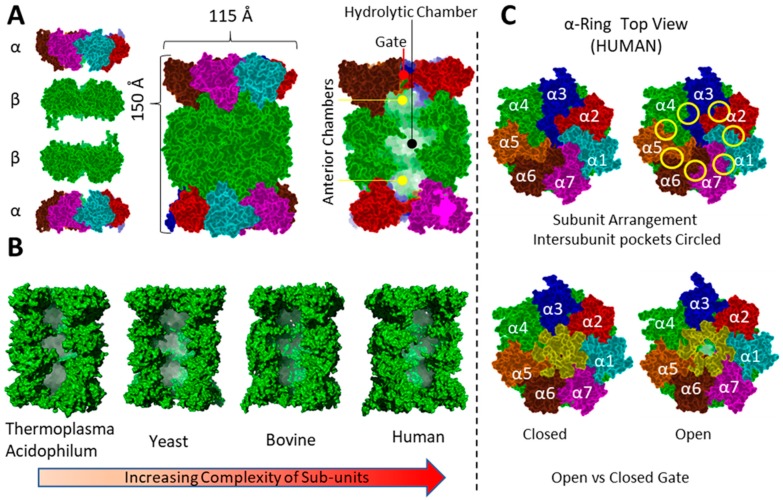
(**A**). Common features of all known 20S proteasomes, illustrating the positioning of the α and β rings, gate, anterior chambers and hydrolytic chamber. (**B**). Comparison of proteasomes, thermoplasma acidophilum, yeast, bovine and human. (**C**). Top view of alpha rings with (in counterclockwise direction) labeled subunits, circled intersubunit pockets, last twenty amino acids colored yellow in open gate, and last twenty amino acids color yellow in the closed conformation.

The quaternary structure of the proteasome is highly conserved (Figure 4) across all known species and consists of four heptameric stacked rings in an α_7_β_7_β_7_α_7_ pattern leading to a hollow cylinder. The completed proteasome is termed the 20S core particle (CP) and is the main protease in use by the cell. This structural arrangement produces three distinct chambers: two anterior chambers at either end and the central hydrolytic chamber. In all cases, the active sites are housed within this chamber along the walls of the β-subunits, in the hydrolytic chamber (Figure 4A). Despite the overall structural similarity, complexity of the constituent subunits greatly increases, going from archaeon to eukaryotic forms. For example, 20S CP from *Thermoplasma Acidophilum* consists of just two unique subunits (α,β) with 14 copies each yielding D7 symmetry [161]. However, the eukaryotic proteasome is comprised of two copies of 14 unique subunits yielding what is termed pseudo-C_2_ symmetry and is true for yeast [162], bovine [163], and human CPs [164]. Sequence similarity drastically changes between yeast and human with a primary sequence of <5% similarity, while the mouse proteasome is reported to have >90% sequence similarity to human. 

The first solved X-ray crystal structure of the human constitutive proteasome was reported in 2015 by Harshbarger and co-workers [164]. Harsbarger et al. obtained high purity constitutive proteasome from red-blood cells that illustrated great structural similarity to previously reported crystal structures of bovine and mouse [165]. However, the authors did note key residue differences that account for differences in site selectivity of certain inhibitors. This suggests that even minor differences among species should be kept in mind when designing and evaluating proteasome regulators in model systems (mouse/bovine models). 

### 3.2. Function of the Proteasome

Eukaryotic proteasomes are threonine proteases which utilize N-terminal threonine residues of three beta subunits to hydrolyze peptide bonds, displaying 3 types of selectivities:

- β1: peptidyl-glutamyl-peptide hydrolyzing (more commonly referred to as caspase-like) and cleaves after acidic amino acids
- β2: trypsin-like activity and cleaves after basic amino acids
- β5: chymotrypsin-like activity cleaving after hydrophobic amino acids.

The human proteasome only presents 6 active sites, two sites per selectivity. Each site is named after the subunit it is housed in (i.e. β1 activity is the activity of the β1 subunit). This combination of multiple catalytic activity coupled with site leeway ensures the rapid degradation of any protein entering the hydrolytic chamber. The mechanism of eukaryotic proteolysis was recently revised by Huber and co-workers to depend on the concerted action of three conserved amino acids (termed catalytic triad) [166]. 

Rampant degradation of cellular contents is prevented by the alpha rings (Figure 4C). Each of the seven α-subunits’ N-termini meet over the central axis of the proteasome and create a physical barrier, termed gate, between cellular contents and the proteolytically active sites. Labeling the CP alpha ring sequentially counterclockwise is the generally accepted method of labeling in the literature (Figure 4C).

A dynamic equilibrium exists between the open and closed gate conformations of the proteasome (Figure 4C bottom). Current information suggests a 3:1 ratio favoring the closed form is likely in a population of free 20S [167,168,169]. However, the opened gate is too narrow to allow properly folded proteins access to the hydrolytic chamber. Only IDPs and oxidatively damaged proteins are natively targeted as they are the only species able to transverse the open gate unaided [152]. More structure species, such as less damaged oxidized proteins, likely require interaction with protein activator proteins (PA) to facilitate passage through the alpha ring gate.

### 3.3. Proteasome Distribution and Aging

It is worth noting that multiple proteasome subtypes exist beyond the “standard” constitutive proteasome described in the preceding section. Each proteasome type may be bound by different activator proteins which dictate that proteasome’s role in the cell: spermatogenesis, DNA repair, general proteolysis, etc. A recent review details these subpopulations [170]. Despite this wealth of diversity, proteolytic clearance of aberrant proteins is handled by two main pathways: the ubiquitin dependent proteasome system (UPS) and the ubiquitin independent proteasome system (UIPS). 

#### 3.3.1. The UPS

Proteins that require proteasomal degradation are marked for it through the conjugation of several ubiquitin (Ub) proteins in a hierarchical sequence involving the action of three enzymes namely, the E1 ubiquitin-activating enzyme, the E2 ubiquitin-conjugating enzyme, and the E3 ubiquitin ligase [171]. Ubiquitin is a 76-residue protein unique to eukaryotic cells with high levels of conservation. Gly76 is activated in an ATP dependent manner by the E1 ubiquitin-activating enzyme forming a thioester linkage with the E1 cysteine. The ubiquitin-conjugating enzyme (E2) binds the Ub-E1 complex and transfers the Ub onto itself through trans(thio)esterification resulting in an E2-Ub complex. Finally, an E3 ubiquitin ligase transfers its bound target protein (most commonly through a lysine residue) to the E2 bound Ub and releases the Ub-protein complex back into the cell, ending the Ub cascade [149]. The cascade is hierarchical with the two E1 enzymes able to bind to multiple E2 enzymes (humans have 35) and each E2 is able to bind to multiple E3 ligases (humans are estimated to have between 500–1000). This tiered system buried within a cascade allows tight regulation of Ub and ubiquitinylated systems. Even though this process is critical to the function of UPS [172,173], a polyubiquitin chain does not guarantee proteolytic destruction, and emerging evidence suggests that the spatial arrangement and linkage specific conformations direct tagged protein outcome and is utilized by the cell for transient post translational modifications (PTMs) [149,171]. 

The canonical PA of the ubiquitin proteasome system (UPS) is the 19S (or PA700), a 700 kDa complex that caps the α-rings of the 20S core particle (CP). 20S CP may be singly (26S) or doubly capped (30S). It should be noted that most literature references inadvertently refer to the doubly capped proteasome (19S-20S-19S), as the 26S proteasome. The 19S is commonly divided into two large segments: a “base” and “lid”. The lid houses recognition sites that bind Ub and draw in labeled proteins [174,175,176,177]. De-ubiquitylating enzymes on the lid release Ub back into solution before other subunits unwind and translocate the protein into the proteasome for processing. 

For details on the ubiquitin dependent proteasome degradation processes readers are directed to several excellent reviews [149,178].

#### 3.3.2. The UIPS

Protein degradation by the 20S CP in the absence of ubiquitin is referred to as the ubiquitin-independent proteasome system (UIPS). It should be noted that a single capped proteasome (19S-20S) still has an open alpha ring allowing Ub independent degradation [179]. However, the contribution of this complex to the UIPS is still unclear and for simplicity, we will restrict our discussion to two main PA of the UIPS: PA200 and PA28. 

As mentioned, the 20S CP does have very low background activity that is enhanced by binding specific PAs. PA200 is a large (200 kDa) monomeric protein that utilizes a conserved C-terminal hydrophobic-tyrosine-any amino acid (Hb-Y-X) recognition feature (vide infra) in a similar manner to the 19S; however, it is only found within the nucleus of mammalian cells [180]. The other PA, PA28 (or 11S or REG), is found in two distinct forms: one in the cytosol, and one in the nucleus. Cytosolic 11S is a heteroheptamer of alpha and beta subunits, while a homoheptamer of a gamma subunit is primarily found in the nucleus. PA28 adopts a toroid shape and binds in the absence of the aforementioned HbYX recognition feature. [181,182]. Additionally, REGs lack the unfolding activity found in the 19S regulatory particles. Instead, upon binding, they induce conformational changes to open the alpha ring gate and increase the flux of suitable substrates through the proteolytic chamber [183]. This mechanism of action precludes properly folded substrates and specifically targets oxidatively damaged and intrinsically disordered proteins. 

In addition to the 20S caps, there are other proteins involved in the regulation of 20S CP-mediated degradation of IDPs, which include NAD(P)H: quinone oxidoreductase 1 and 2 (NQO1 and NQO2), the heat shock protein 90 (HSP90) and Poly [ADP-ribose] polymerase 1 (PARP-1). [184,185,186,187,188,189,190]. The detailed mechanism by which these proteins regulate 20S mediated degradation of IDPs is still largely unclear. 

While both the UPS and UIPS may target IDPs for degradation (a specific example of UIPS targeting IDPs is p53 and p73 [187] and other evidence is available [191,192,193,194,195]), the UPS targets structured and misfolded proteins in need of removal, [151,196,197,198] whereas the UIPS is restricted to the removal of oxidatively damaged and disordered proteins. 

#### 3.3.3. Mechanism of Proteasome Gating

The preponderance of evidence has prompted investigation into proteasome activation as a potential therapeutic strategy for clearance of pathogenic proteins, most notably in neurodegenerative diseases [152,199,200,201,202,203,204]. For example, purified 20S introduced to cells via direct injection showed accelerated clearance of tau [205]. Tau aggregation was also diminished when mutant 20S was expressed lacking the N-termini of alpha substituents (i.e. an ungated 20S). 

Therapeutically this strategy seeks to take advantage of the latent 20S pool found in older individuals [206,207] by mimicking the effect of endogenous PAs. Understanding the mechanism of gating is of paramount importance to this endeavor. The ideal case would undoubtedly be mimicking the activity of an UIPS PAs as they are thought to operate via pure conformational action and allow enhanced degradation (up to 20x) independent of other species (i.e. ATP, Ub, etc.) [208]. 

### 3.4. The 11S and Proteasome Binding

**Figure 5:**
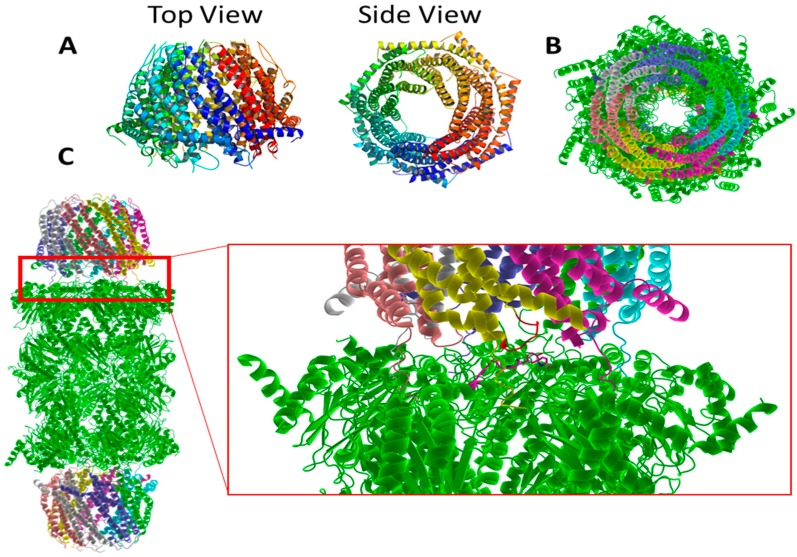
(**A**). 11S activator with multi-colored monomers. (**B**). Top view of 11S-20S-11S yeast proteasome complex. (**C**). Side View of 11S-20S-11S yeast complex. Red Box zoom callout illustrating the 11S-20S interaction. Magenta residues illustrate conserved recognition sequence of Tyr8, Asp9, Pro17, and Tyr26 interacting beneath the yellow 11S subunit. Activation loop colored red on 11S.

The 11S (also known as REG or PA28) is involved in processes related to the immune response [209,210]. Higher eukaryotes express three isoforms termed α,β, and γ. PA28α and Paβ form a heteroheptamer (Figure 5A) while the γ isoform is a homoheptamer [210]. It is important to note that most of the data on 11S mediated mechanism of gating has been collected via analysis of yeast proteasome complexes, and may not be completely analogous to human structures. 

The structure of human REG28 has been known for twenty years [211]. The α monomer has a relative mass of 28,700 Da in a predominantly helical configuration. A disordered 39 residue loop distinguishes different REG isoforms from one another. The mechanism of gating has been elucidated for PA26 (the yeast version of human REG28) in complex with proteasomes from *S. cerevisiae* [180,183,212] and *Thermoplasma acidophilium* [212]. C-terminal residues form main-chain to main-chain hydrogen bonding and a salt bridge between the C-terminal carboxylate and Lys 66. Interaction with the Pro17 reverse turn on the proteasome with an “activation loop” (Figure 5C) on PA26 induces gate opening by small (0.5–3.5 Å) movements of each subunit. Four conserved residues were identified as crucial to binding and stabilization of the open form: Tyr8, Asp9, Pro17, and Tyr26 [180,213]. Severe reduction in model substrate degradation was observed in mutant archaeal proteasomes with modifications to any of these residues. 

**Figure 6:**
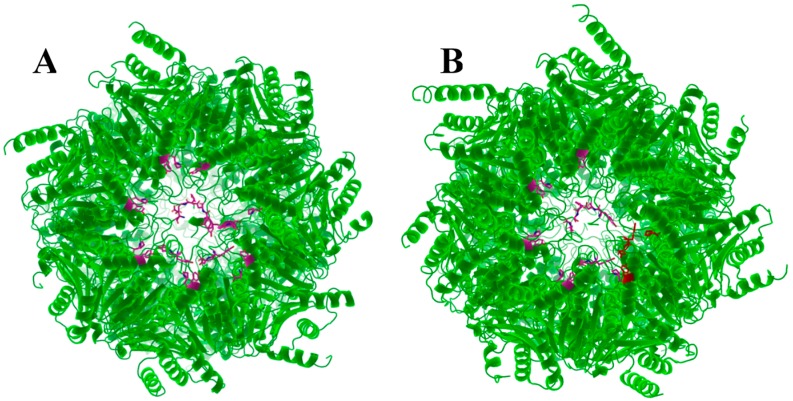
(**A**). Top view yeast 20S proteasome with the recognition sites highlighted magenta. (**B**). Top view of the human 20S proteasome with recognition sites colored magenta and similar residues in red.

In yeast, these conserved sequences are present on every α-subunit (Figure 6A). However, analysis of recent crystal structures of the human 20S proteasome reveal that two subunits (α1 and α2) completely lack this recognition motif (Figure 6B). Four subunits (α3, α4, α5, and α6) present the conserved motif in the proper relationship (i.e. Tyr8, Asp9, then 8 amino acids to Pro17, then 9 amino acids to Tyr26; Figure 6B magenta residues). The final subunit, α7, provides the four near one-another but in a highly varied spatial arrangement (Figure 6B red residues). The consequences of human α7’s varied presentation are currently unknown.

### 3.5. The 19S 

Due to the distinct influence of 19S capped proteasome within the UPS system, great effort has been put into understanding the dynamics of this complex. Recent reviews discuss many aspects of the 19S-20S holoenzyme [214] (26S proteasome) and will not be detailed here. Rather, we will outline the 19S and focus on key aspects of binding and gating mediated by the 19S regulatory particle (RP). Excitingly, the last few years have brought great insight into this mechanism in *homo sapiens* samples and shown the process to be quite complex.

**Figure 7:**
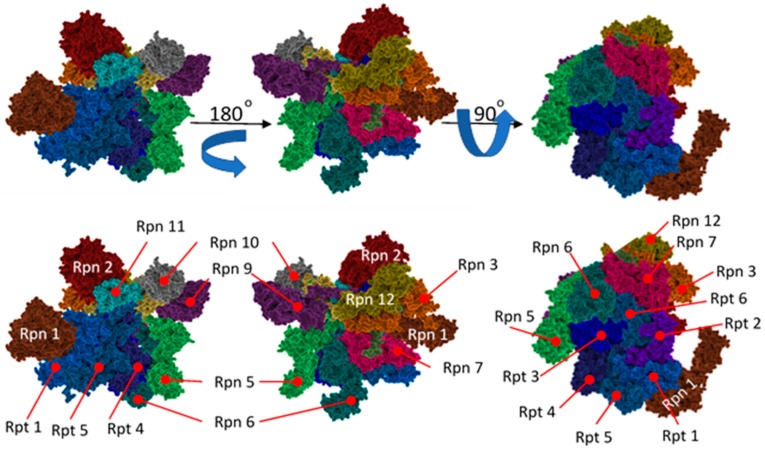
Surface representation of the 19S protein activator with individual subunits labeled. The “base” is depicted in shades of blue while the “lid” is presented in multiple colors.

The 19S is divided into to two parts: the base and the lid. The lid houses several subunits responsible for various functions (Figure 7). Rpn1, Rpn10, and Rpn13 (not shown) recognize ubiquitinylated substrates [158,214], Rpn11 is a zinc dependent deubiquitinase (DUB), while other Rpns offer structural support and aid cooperatively in the unfolding of bound proteins. 

This process is regulated by the motor units that make up the base. Six distinct subunits (Rpt1–6) from the AAA (ATPases associated with diverse cellular activities) family of ATPases regulate lid mediated engagement and unfolding [148,157]. This process is conformationally complex as the holoenzyme has been reported to exits in 19 distinct states [214,215,216,217]. Two works of special note are those of Dong et al., who published [218] a study on the dynamics of substrate-engaged human 26S using Cryo-EM, and a work by Wang et al. [219], who utilized cross-linking mass spectrometry (XL-MS) to identify nearly 100 inter and intra protein interactions leading to multiple new conformations and dynamic regulations. Interested readers are referred to those publications for details of 19S conformers, as we will focus exclusively on interactions directly related to the 20S binding and gating.

### 3.6. Hb-Y-X Motif 

A conserved C-terminal hydrophobic-tyrosine-any amino acid (Hb-Y-X) motif triggers 20S gate opening upon ATP binding, and is found on a number of proteasome binding partners including assembly factors [220] and activator proteins [221]. One of the first studies of the importance of the HbYX motif was done by Smith et al. using PAN and archaeal 20S [221]. Systematic mutational studies demonstrated that a hydrophobic residue and then penultimate tyrosine were critical for proteolysis. However, the terminal amino acid had a degree of variability (Table 1). 

The authors went on to show that no AA substitution was tolerated at the penultimate tyrosine. Additionally, they discovered that lys66 within the α-subunit was needed for PA-20S association. Based on this information the authors concluded that the penultimate tyrosine and a preceding hydrophobic residue were essential for gating, but the terminal AA was only required for PA association and played no role in substrate hydrolysis. Incidentally, lys66 is also the anchor point for the 11S though it does not express the HbYX motif (see Section 3.3). 

For nearly twenty years, studies such as these have demonstrated the importance of the HbYX motif in the endogenous gating mechanism and it was believed that the binding of the HbYX motif was sufficient to induce gate opening (corroborated by several studies on peptide mimics possessing HbYX tails) [221,222]. However, recent Cryo-EM studies have revealed the stable binding of eukaryotic Rpt2, 3, and 5 in 26S structures with a closed gate proteasome [167]. Over the interim years, many groups have pursued similar studies to fully determine the mechanism of 26S degradation [217,219,223,224,225]. Recently, the human 26S holoenzyme has been solved to a high resolution, and has been reported to exist in at least 11 unique states: four in the absence of substrate and seven in the presence of substrate [215,218,219]. 

### 3.7. Gating of the Proteasome

In the absence of substrate engagement, the 26S’s four states have been termed A (ground state), B (the commitment), C (gate priming), and D (gate-open) states [218]. Interestingly, the ground state, when challenged with ATP-γ-S, exhibits an asymmetrically open-gate, wherein, the open gate is opposite to the 19S binding. The substrate engaged D state (S_D_ state) likewise exhibits asymmetry but has the open gate on the end bound by 19S [215]. Analogous states have been identified in yeast and are termed S_A_, S_B_, S_C_, and S_D_ respectively. In a more recent publication [218], substrate engaged human 26S has been described in 7 different states: termed E_A1_, E_A2_, E_B_, E_C1_, E_C2_, E_D1_, and E_D2_. Excellent work published on 26S dynamics [159,179,206,218,226] allows for great understanding of substrate recognition, unfolding, and translocation events; however, due to pocket geometry, a variety of PA-CP interactions are available, resulting in a limited mechanistic understanding of which residues are key to generate an open gate CP. Among the 11 states listed above, only the C-termini of Rpt3 is generally unchanged, whereas Rpt5 goes through minor conformational changes, and Rpt1, 2, and 6 display high variability between states. 

**Figure 8:**
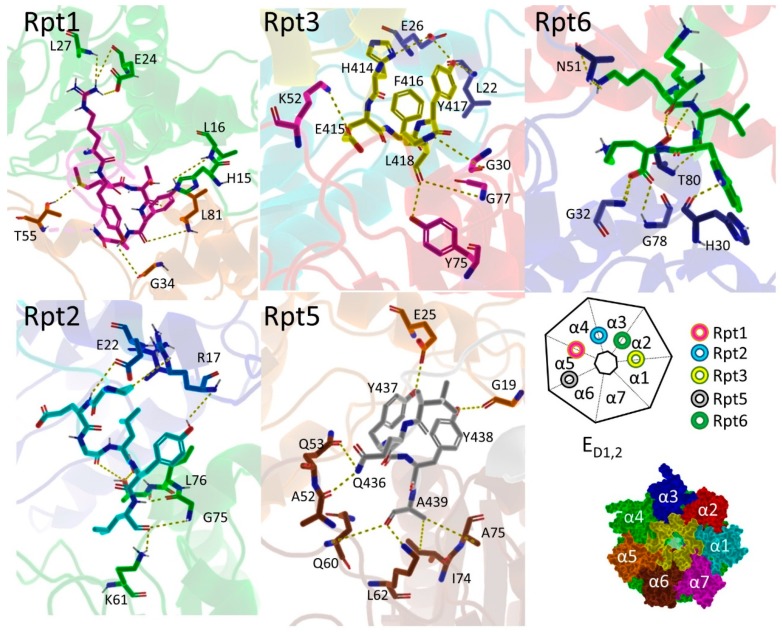
Suggested residues of interaction between C-termini of 19S base and CP alpha units in the E_D2_ open state. Schematic representing where Rpt tail insertions are occurring and are colored coordinated with the crystal structure images shown.

These new data imply that the Rpt3 operates as an anchor for the 19S base, as strong hydrogen bonding interactions are present at multiple points along the bottom of the α2 subunit and the front of the α1 (Figure 8). These interactions are preserved across most of the other solved crystalline forms despite large conformational changes in the lid and minor changes in the alpha ring geometry. This binding dynamic is the same in the other HbYX containing base subunits Rpt2 and 5 (i.e., hydrogen bonding network along the back and bottom of one α-subunit and front of the adjacent α-subunit). The Rpt5 displays minor conformational changes through the substrate processing process, while the Rpt2 possesses more varied conformations while preserving the number of contacts, if not the same contacts (Figure 8). 

However, Rpt1 and 6, lacking the HbYX motif, begin changing this commonality. Rpt1 binds most similarly to Rpt2, 3, and 5; however, it also bridges off to attack more centrally residing AAs within the α4 subunit by making a salt bridge with E26 and accepting a hydrogen bond from L27. 

In the case of Rpt6 binding, the multi-subunit binding breaks down completely, as only minor hydrophobic interactions appear to be present with the front of α2, as Rpt6 instead opts for numerous interactions with the α3 subunit. The α3 subunit is often cited as the most important subunit in gating, as it possesses the greatest amount of electron density over the CP opening. Perhaps this extreme binding interaction with the Rpt6 tail is required to induce the conformational swing that moves the N-termini away from the CP opening; however, no clear indication of how this is achieved is currently available. 

**Figure 9:**
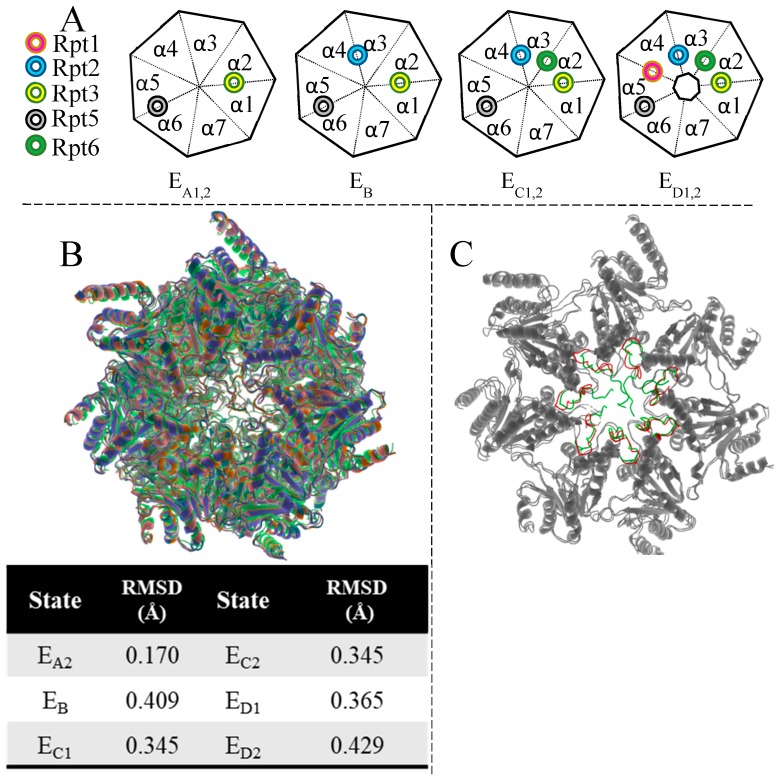
(**A**) Schematic representation of Rpt units inserting C-termini into acceptor pockets on the 20S face. The Cp is represented as a heptagon and the Rpt tails as colored tori. (**B**) Image created from deletion of 19S subunits from deposited pdb structures corresponding to the listed states (pdb id: 6MSB, 6MSD, 6MSE, 6MSG, 6MSH, 6MSJ, 6MSK respectively) then alignment of all to the CP structures of 6MSB. Unbound free proteasome CP (4R3O) also aligned to 6MSB. Table values recorded from pymol output after completion of alignment. (**C**) Alpha Rings of E_A1_ overlaid with E_D2_. N-termini colored green in E_A1_ and red in E_D2_.

The limited understanding of factors effecting gating is perhaps best illustrated by the fact that despite diverse conformational changes occurring in the 19S system, the 20S CP is largely unchanged (Figure 9). Deletion of the 19S subunits and alignment to a single structure (E_A1_, Figure 9B) shows incredible cohesion among these seven states with highest relative mean standard deviation (RMSD) found in the final open form (0.429 Å). As the holoenzyme progresses through these states, base Rpt units engage intersubunit pockets on the 20S surface (Figure 9A), yet provoke little diversity in the CP. Indeed, between a gate open form and a closed gate 19S bound CP (Figure 9C), only the last 20 AA appear to make major conformational changes. 

The uncertainty in the above discussion highlights the need for more detailed understanding of PA-CP interactions leading to the open-gate state. The size and complexity of this problem has made even the identification of non-protein agonists a challenging task. In the next section, we will discuss the current state of the field in identifying and manipulating proteasome agonist and highlight current challenges to future progress. 

## 4. Proteasome Activators

### 4.1. Denaturants

Sodium dodecyl sulfate (SDS) was the first reported agonist of the 20S proteasome. Proteasome activity can be measured by the release of AMC from the fluorogenic chymotryptic-like (CT-L) peptide substrate (SUC-LLVY-AMC) over time. Hydrolysis of SUC-LLVY-AMC was stimulated 20-fold above baseline using SDS; however, this could only be achieved above SDS’s critical micellar concentration, suggesting that a detergent-like activity is the probable mechanism of action. Systems which induce activity in this way are suggested to partially denature alpha ring subunits, allowing passage of substrate in the hydrolytic chamber [227,228]. Activation at low concentrations and inhibition at higher concentration, i.e., a narrow range of activity, is a common hallmark of detergent-like species [229]. A number of compounds have exhibited this behavior, including polycations, polyanionic lipids [230], fatty acids [231], and a natural product oleuropein [232]. Compounds of this class are generally avoided due to the likelihood of non-specific interactions and complex mechanisms which limit their ability to be optimized. 

### 4.2. Peptides 

The most common class of proteasome gate openers are synthetic peptides based upon the HbYX motif. Activity of these vary greatly depending on which PA they are modeled after, yet several have been reported to increase turnover of oxidized [233] proteins [234,235,236]. Recently Giżyńska et al. disclosed work on peptides that could be tuned as either proteasome agonist or antagonists [237]. Low micromolar activities can be achieved which mimic this behavior; however, intrinsic challenges remain. In general, peptide mimics suffer from low metabolic stability and poor membrane permeability [233]. Despite this, synthetic peptides make excellent probe compounds due to often straightforward, though non-trivial, synthesis. 

### 4.3. Small Molecules

#### 4.3.1. Direct 20S Activators 

**Figure 10:**
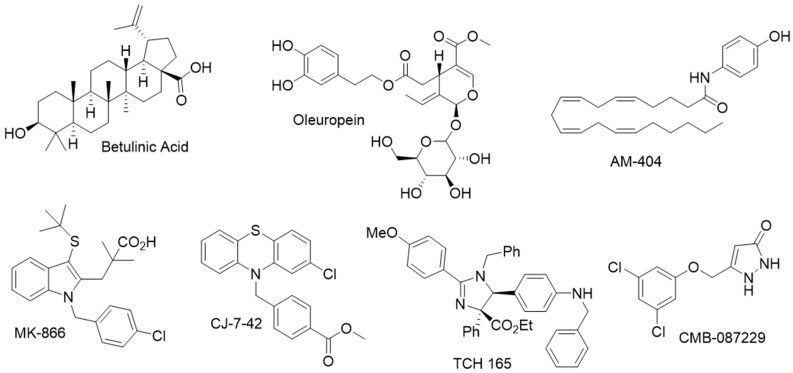
Selected examples of known proteasome agonist demonstrating the structural diversity.

Betulinic acid is an early report of a small molecule capable of increasing proteasome activity (Figure 10). A triterpene natural product, betulinic acid, selectively enhances the chymotryptic-like site of proteasome activity. Synthetic modifications yielded inhibitory analogs, suggesting a complex structure activity relationship (SAR) [238].

Testing of proteasome agonists and antagonists is typically carried out in vitro by monitoring the rate of hydrolysis of an idealized fluorogenic peptide substrate that displays selectivity for a particular active site [3,239,240,241]; the most often used is the chymotryptic selective succinyl-Leu-Leu-Val-Tyr-7-amino-4-methyl coumarin (SUC-LLVY-AMC). The convenience of this method makes it very amenable to high throughput screens (HTS). However, some lament the non-physiological system and question its ability to translate to cellular action. 

The Kodadek lab proposed that some compounds may open the 20S gate enough to allow increased turn-over of the relatively small fluorogenic peptides, but not wide enough to allow larger misfolded proteins to be degraded [229]. They investigated this by performing a HTS of NIH Clinical Collection (NIH NCC), which identified 12 primary hits. Using these compounds, they developed a series of follow-on assays [229]. Hit compounds were then subjected to a quantitative LCMS assays to determine each compound’s ability to degrade protein substrate. Efficacious compounds were then tested for their ability to stimulate the proteasome in HEK cells using green-fluorescent protein monitoring. This three-step validation method yielded two bona fide proteasome agonists: MK-866 and AM-404 (Figure 10). Recently, Trader’s lab published an SAR study on AM-404 illustrating chain-length importance and, more strikingly, on the necessity of a *cis*-alkene at C8 for enhancing 20S proteasome activity [242]. Although this work did not lead to a greatly improved agonist, it did improve toxicity over the original lead while preserving stimulatory activity. 

HTS have been the preferred method of hit-to-lead campaigns. Trader developed a fluorescence resonance energy transfer (FRET) reporter assay for the HTS of proteasome activators [243,244]. The much larger size of the FRET reporter lowered the basal rate of hydrolysis and provided higher sensitivity and increased dynamic range over the field standard peptides. Using this new system, they identified 3 new proteasome agonists from an HTS of 800 compounds. The mechanism by which these leads activate the proteasome is still not known.

Our own lab screened the NIH Clinical Collection (NCC) and Prestwick libraries, which found the neuroleptic chlorpromazine as a potent proteasome agonist inducing up to 20-fold activity in vitro [245]. In a dual effort to remove dopamine activity while preserving proteasome agonism, we utilized an unbiased in silico docking screen to locate potential binding sites within the 20S CP. Possible interactions were predicted within proteasome intersubunit pockets in a similar manner to the HbYX motifs, suggesting an intrinsic allosteric mechanism. Chemical modification of chlorpromazine successfully abrogated its dopamine activity, while retaining low micromolar potency in activating the 20S proteasome (Figure 10; CJ-7-42).

Although the chlorpromazines are suggested to operate via inducing an open gate proteasome, strictly speaking, the only currently known small molecule empirically shown to cause such a dramatic conformational change is the imidazoline TCH-165 (Figure 10) [246]. Recently we disclosed this compound’s intriguing ability to modulate the proteasome assembly via the induction of an open gate conformer. AFM imaging illustrated that the ratio of open to closed 20S CP was affected in a dose-dependent manner and correlates with the increased activity of all three proteolytic active sites. TCH-165 prevents binding of the Rpt3 peptide and the 19S RP to the 20S CP, suggesting a direct binding to the α-ring. Consistent with this, TCH-165 does not enhance the fully assembled proteasome (19S-20S-19S), which has the alpha-ring blocked by the two 19S caps. A definitive binding site is still unknown, though docking simulations strongly suggest binding in the hydrophobic α2-α3 intersubunit pocket. 

#### 4.3.2. Indirect Proteasome Activators

Pyrazolone (Figure 10, CMB-087229) was identified to be a small molecule activator of the proteasome and showed significant disease attenuation in vivo models of amyotrophic lateral sclerosis [247]. Affinity pull down experiments verified its association with the proteasome, but the mechanism of its regulation is also still unclear.

The proteasome also undergoes various post-translational modifications, including phosphorylations, that affect its cellular activity [201]. Leestemaker et al. identified several p38 MAPK inhibitors that indirectly enhanced proteasome activity, resulting in an increase of the survival of cells over expressing α-synuclein [248].

The cAMP-dependent protein kinase A induces proteasome activity. Consequently, increasing c-AMP-PKA signaling using the phosphodiesterase type 4 inhibitor, Rolipram, resulted in enhanced proteasome activity, reduction of tau aggregation and improved cognition in vivo [249]. 

Upregulation of proteasome activity can also be achieved through activation of the transcription factor (Nuclear factor (erythroid-derived 2)-like 2 (Nrf2)). The antioxidant 3*H*-1,2-dithiole-3-thione (D3T) upregulates both 20S and 19S proteasome subunits, resulting in an increase in proteasome activity, though only in Nrf2 positive fibroblasts [250]. 

Hedstrom and co-worked applied a clever approach where a tert-butyl carbamate (Boc_3_A)-protected arginine linked to a covalent inhibitor of glutathione transferase (GST) induced the degradation of glutathione transferase (GST-α1) in an ubiquitin-independent manner by the 20S proteasome [251]. 

The inhibition of deubiquitinase (DUB) activity has also become an exciting new field of research as an indirect method to manipulate ubiquitin dependent proteolysis. For example, Lee utilized HTS to identify a selective inhibitor of USP14, an endogenous proteasome inhibitor, which increased protein turnover in cells by preventing USP14 mediated chain trimming [252]. This exciting new area of increasing protein degradation has been reviewed extensively, and readers are referred to those excellent reviews [253,254,255,256]. 

### 4.4. Conclusions 

There are strong similarities between oxidatively damaged proteins and IDPs. The ubiquitin-dependent and -independent proteasome-mediated clearance of those disordered targets is required to maintain healthy proteostasis in the cell. A decrease in proteasome activity and/or accumulation of disordered proteins may result in proteotoxic stress, which is observed in multiple neurodegenerative disorders. Proteasome agonists mimic endogenous PA activity, restore proteasome function, and return homeostasis. 

However, before this may be realized, much work remains to be done. Currently, the long-term effect of proteasome enhancement in cells is still unknown. Much work is needed on deciphering the mechanism of proteasome gating to aid SAR campaigns, and investigations into determining the minimum activity enhancement required to produce cellular modifications are needed. However, the diversity of structural features (Figure 10) and the continued improvements in assay design show great promise for future research in this exciting field. 

## Figures and Tables

**Table 1:** Mutation Study from Smith et al.

Terminal Sequence	Hydrolysis (%WT)
LYR (WT)	100
LY-	4
LYD	5
LYA	100
LYW	106
LYL	13
LYG	77
YRA	2
No PA	5
